# The effect of hip abductor fatigue on knee kinematics and kinetics during normal gait

**DOI:** 10.3389/fnins.2022.1003023

**Published:** 2022-10-04

**Authors:** Yuting Tang, Yanfeng Li, Maosha Yang, Xiao Zheng, Bingchen An, Jiejiao Zheng

**Affiliations:** ^1^Department of Rehabilitation, Municipal Hospital of Traditional Chinese Medicine Affiliated to Shanghai University of Traditional Chinese Medicine, Shanghai, China; ^2^Department of Rehabilitation, The Second Rehabilitation Hospital, Shanghai, China; ^3^Department of Rehabilitation, HuaDong Hospital, FuDan University, Shanghai, China

**Keywords:** hip abductor, fatigue, knee joint, kinematics, kinetics

## Abstract

**Objective:**

To investigate the effect of hip abductor fatigue on the kinematics and kinetics of the knee joint during walking in healthy people to provide a new approach for the prevention and treatment of knee-related injuries and diseases.

**Methods:**

Twenty healthy participants, ten females, and ten males, with a mean age of 25.10 ± 1.2 years, were recruited. Isometric muscle strength testing equipment was used to measure the changes in muscle strength before and after fatigue, and the surface electromyography (SEMG) data during fatigue were recorded synchronously. The Vicon system and an AMTI© force platform were used to record the kinematic parameters and ground reaction force (GRF) of twenty participants walking at a self-selected speed before and after fatigue. Visual 3D software was used to calculate the angles and torques of the hip and knee joints.

**Results:**

After fatigue, the muscle strength, median frequency (MF) and mean frequency (MNF) of participants decreased significantly (*P* < 0.001). The sagittal plane range of motion (ROM) of the knee (*P* < 0.0001) and hip joint (*P* < 0.01) on the fatigue side was significantly smaller than before fatigue. After fatigue, the first and second peaks of the external knee adduction moment (EKAM) in participants were greater than before fatigue (*P* < 0.0001), and the peak values of the knee abduction moment were also higher than those before fatigue (*P* < 0.05). On the horizontal plane, there is also a larger peak of internal moment during walking after fatigue (*P* < 0.01).

**Conclusion:**

Hip abductor fatigue affects knee kinematics and kinetics during normal gait. Therefore, evaluating hip abductor strength and providing intensive training for patients with muscle weakness may be an important part of preventing knee-related injuries.

## Introduction

Knee injuries, such as anterior cruciate ligament (ACL) injuries and patellofemoral pain syndrome (PFPS) (Hutchinson and Ireland, 1995; Laprade et al., 2019), are the most common sports-related injuries. Knee osteoarthritis (KOA) is the most common knee disease in persons 60 years of age or older, seriously affecting the mobility of the elderly (Sharma, 2021). During weight-bearing activities, the knee joint carries the greatest load and therefore has the potential for injury to the joint (Kumar et al., 2013). Although the occurrence and development of ACL injury, PFPS, and KOA are caused by a combination of multiple, abnormal knee biomechanics in the coronal plane appears to be a common risk factor for disease factors (Moyer et al., 2014; Montalvo et al., 2019; Neal et al., 2019). The focus of the current studies was on the external knee adduction moment (EKAM), which is determinative of medial knee joint loads (Landry et al., 2007; Foroughi et al., 2009; Duffell et al., 2014). Previous studies have shown that the higher EKAM seen in females compared to males may suggest an increased risk for the development of KOA in females with ACL-reconstruction (Webster et al., 2012). Patients with medial compartment knee osteoarthritis (MC-KOA) or PFPS usually have a higher EKAM (Bolgla et al., 2008; O’Connell et al., 2016), and the peak EKAM is positively correlated with disease progression and increased pain (Birmingham et al., 2019; Rees et al., 2019). However, recent studies have shown that hip abductor also play an important role in those diseases (Willy and Davis, 2011; Bell et al., 2016).

As the proximal joint of the lower limbs, the hip joint plays an integral role in maintaining balance and providing stability (Pel et al., 2008). However, this function depends on the hip muscles providing dynamic stability during exercise (Williams et al., 2001). As a result, hip muscle weakness may lead to certain movement dysfunctions, putting certain muscles and joints, especially the knee joint, at high risk of injury. In recent years, some scholars have reported that the weakness of hip abductor (such as gluteus medius) may be an important reason for the increase in EKAM (Kean et al., 2015; Bennett et al., 2018). The hip abductor mainly stabilize the femur in the frontal plane of the lower limb movement (Mcleish and Charnley, 1970). The weakness of hip abductor makes the hip more prone to adduction or rotation during weight-bearing activities (such as jumping or landing). Abnormal hip movements increase the abduction angles and moments of the knee joint and affect the muscle activation of the hip abductor and quadriceps femoris. The shearing force exerted on the tibia by the quadriceps during jumping or landing can increase the abduction angle of the knee or abduction moment of the knee and abnormal muscle activation, which further increases the tension of the ACL and increases the risk of ACL injury (Hewett et al., 2005). Studies have shown that most patients with PFPS have decreased strength in the hip abductor muscles (gluteus maximus and gluteus medius) (Magalhaes et al., 2013; Rathleff et al., 2014; Neal et al., 2019). Contralateral pelvic drop and internal rotation of the femur are the most common methods for improving defects in the abductor muscle of the hip, which presumably would result in higher peak EKAM to further affect the occurrence and generation of PFPS (Bolgla et al., 2008; Baldon et al., 2009; Powers, 2010).

Patellofemoral pain syndrome patients experience decreased activity and delayed start time in the hip abductor muscles (Gluteus medius, gluteus maximus) when performing tasks such as standing on one leg, squatting, or running (Willson et al., 2011, 2012; Mirzaie et al., 2019). Fukuda et al. (2010) and Lack et al. (2015) demonstrated that hip abductor strength training can relieve pain and improve the function of patients with PFPS, supporting the theory that hip abductor have an effect on PFPS to some extent. Deasy et al. (2016) showed that the strength of hip abductor muscles in symptomatic KOA patients was significantly weaker than that in healthy controls. Greater external hip abduction moments while walking have been shown to reduce the risk of KOA progression (Chang et al., 2005). Therefore, we hypothesized that decreased hip abductor strength may lead to an increased load in the medial compartment of the knee joint.

However, it leaves doubt whether the weakness, insufficiency and activation pattern changes of the hip abductor existed before injury or became evident secondary to the injury. Previous studies used fatigue protocols to simulate hip abductor deficiency and then measured the effects of knee kinematics and kinetics to identify a cause-and-effect relationship. However, because the fatigue degree of patients has not been strictly quantified and the effect of fatigue recovery has not been considered, the conclusion of the study on the effect of hip abductor fatigue on the knee joint is disunified. Patrek et al. (2011) studied the influence of hip abductor fatigue on the single-leg landing biomechanics of female athletes but found no change in the kinematics and kinetics of the knee joint. However, Geiser et al. (2010) found that hip abductor fatigue increased peak EKAM during cutting, jumping, and running. Based on this, we made three attempts to quantify the level of fatigue over the fatigue protocol. (1) Ratings of the Borg perceived exertion scale (on a 6–20 scale) were recorded every 10 s during the exertion protocol; (2) peak hip-abductor strength immediately after the exertion protocol was compared with hip-abductor strength values before the protocol; and (3) muscle activity patterns were measured by surface electromyography (SEMG) over the fatigue protocol.

The purpose of this study was to determine the effects of weakness induced by isolated hip abductor fatigue on lower limb kinematics and kinetics in healthy participants. Specifically, we hypothesized that knee kinematics and kinetics would change after an isolated hip abductor fatigue protocol.

## Materials and methods

### Participants

Twenty healthy participants, 10 females and 10 males, 21–35 years old, were recruited. The sample size was based on previous studies (Geiser et al., 2010; Patrek et al., 2011). Participants were free from any lower limb joint or muscle pain or injury that limited activity in the past 6 months, any neuromuscular condition that precluded exercise training, and any past injury requiring surgery to the lower extremity. All participants provided written informed consent, and the study was reviewed and approved by the ethics committee of Huadong Hospital (No. 2020K079).

### Instrumentation

The kinematics of the hip and knee joints were recorded at a sampling rate of 100 Hz using an 8-camera motion capture system (Vicon^®^ T40, Oxford Metrics, Oxford, UK). One force platform (model OR6-7; Advanced Mechanical Technology, Inc., Watertown, MA, USA) embedded on the laboratory floor captured the ground reaction force (GRF) at 1,000 Hz. A wireless EMG recording system (Telemyo 2400T G2, Noraxon, Scottsdale, AZ, USA) operated at 2,000 Hz was used to synchronously record the EMG of the gluteus medius (GM) muscle. A HYGJTL-002 handheld dynamometer was used to test the maximum isometric strength of the hip abductor.

### Experimental protocol

**FIGURE 1:**
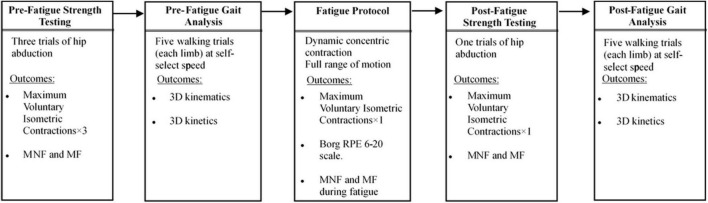
Experimental protocol.

The study protocol is illustrated in Figure 1. The first thing is to familiarize participants with the experimental procedure. Before the test, the dominant leg (leg to be fatigued) was selected for each participant by asking which leg was preferred for kicking the ball. Participants underwent hip abductor strength testing and biomechanical gait analysis before and after unilateral hip abductor fatigue (prefatigue and postfatigue, respectively). Hip abductor strength tests were conducted before gait analysis in the pre-fatigue and post-fatigue condition. After the postfatigue gait, hip abductor strength was collected immediately to determine the level of recovery from fatigue during the gait test.

### Strength testing

The participants completed three series of maximum voluntary isometric contractions (MVIC) of the study limbs on the handheld dynamometer (HYGJTL-002). The position of each participant was as follows: they lie on the side of the non-dominant leg, the dominant leg knee was straight, and the dominant hip flexion, abduction and rotation were 0°. To maintain the consistent placement of the hand-held dynamometer in all strength tests, a marker was placed on the skin near 2.5 cm of the lateral femoral epicondyle of the dominant leg. The handheld dynamometer was placed on this mark in all strength tests. Participants first warmed up with a perceptual maximum of approximately 50% and then performed three maximal effort contractions. Each MVIC trial lasted for 5 s, with a 2-min break between the tests. In each MVIC trial, participants were told not to flex their knees and to keep the toes at the top of their thighs pointing forward to help prevent changes in muscle recruitment and compensation during the test. After the fatigue protocol, the MVIC trial was measured repeatedly using the same scheme, and the MVIC was measured after post-fatigue gait analysis, but only one maximum effort contraction was carried out.

### Surface electromyography

Electromyography activity of the GM muscle was recorded during the first and last 30 s of the fatigue protocol. The skin was wiped and cleaned with 70% alcohol before placing the sensor. Wireless bipolar EMG sensors (Delsys Inc, Natick, MA, USA) were placed on the midpoint of the line between the most cranial and lateral point of the iliac crest and the greater trochantor. We chose the GM to place the sensor because although most studies measure the overall strength of the abductor muscle of the hip joint, reduced strength is usually interpreted as weakness of the GM (Sogaard et al., 2006; Decorte et al., 2012). In addition, the lower limb kinematics related to hip abductor weakness, such as the weight acceptance (WA) portion of the stance phase, hip adduction, internal rotation and knee abduction (Kluger et al., 2013), are consistent with the decrease in activity of the posterior GM muscle (Sogaard et al., 2006; Muotri et al., 2107). Signals use a Trigno TM wireless system with analog-to-digital conversion at 2000 Hz.

### Gait biomechanics procedures

Kinematic data were collected using an 8-camera motion capture system (Vicon^®^ T40, Oxford Metrics, Oxford, UK) sampling at 100 Hz. GRF data were collected at 1000 Hz from one force platform (model OR6-7; Advanced Mechanical Technology, Inc., Watertown, MA, USA) along a 10 m walkway. To monitor joint and segment motion over the ground, 22 reflective markers (14 mm in diameter) were placed on the participant’s anatomical landmarks: the anterior-superior iliac spine (ASIS), posterior-superior iliac spine (PSIS), greater trochanter, lateral and medial femoral condyles, lateral and medial malleoli, posterior portion of the calcaneus, and head of the first, second, and fifth metatarsals. The four anatomical frames were rigid clusters of four on orthogonal markers and were placed on the lateral sides of the bilateral thighs and shank. A static trial was conducted as a reference to determine the body mass and the positions of the joint centers. After the static neutral standing test, the kinematic and kinetic gait data of five lower limb ground and barefoot walking trials were collected. Previous studies have shown that the average fatigue time of abduction fatigue tests is approximately 2∼3 min, so the five walking trials after fatigue were completed within 3 min (Patrek et al., 2011). Walking velocity was monitored using the TC Timing System (Brower Timing System, Salt Lake City, UT, USA) and was maintained within ± 5% of the self-selected speed of the participant.

### Fatigue protocol

**FIGURE 2:**
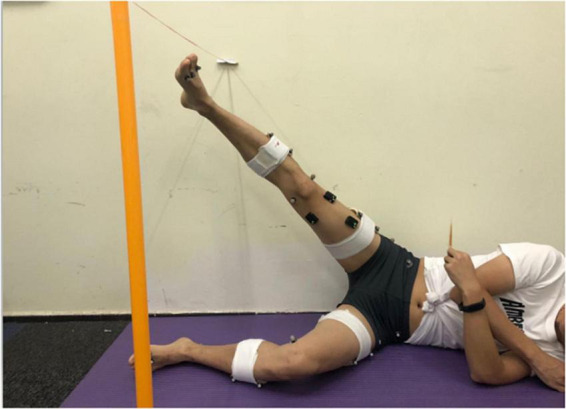
Participant positioning for the hip abductor fatigue protocol.

The hip abductor fatigue protocol used in this study is based on previous studies (Patrek et al., 2011; Soriano-Maldonado et al., 2014). Participants were positioned side-lying on the floor in a starting position of full knee extension and neutral hip position. Participants slowly abducted the hip of the top (dominant) limb while keeping the knee in extension, the tibia and femur in a neutral transverse plane position, with the bottom limb stationary. Participants stopped at 30° of hip abduction and returned to the starting position (the side-lying hip abduction exercises led to the greatest activation of the GM; Distefano et al., 2009). The height of the participants’ hip abduction was marked by a plastic bar at the height of 30° of hip abduction, and the hip abduction angle was measured by a standard goniometer. The bar provided the participant with a fixed goal of hip abduction of 30° and provided tactile feedback for achieving the goal (Figure 2). Each participant was asked to perform hip abductions at a rate of 60 beats per minute (provided by a digital metronome) until she reported a Borg perceived exertion scale rating of 19 or greater (on a 6–20 scale) and failed to reach the plastic bar at the required tempo on two consecutive days. The hip-abduction strength was tested again when the two fatigue criteria were met.

Our study used three methods to quantify fatigue. First, ratings of the Borg perceived exertion scale (on a 6–20 scale) are recorded every 10 s in the fatigue protocol. RPE scores correlate well with both physiological measures of stress and arousal threshold (e.g., HR, ventilatory, blood lactate and creatinine concentration) as well as psychological measures of exhaustion (Soriano-Maldonado et al., 2014). Second, the peak hip abductor strength before and after the exertion protocol was compared because fatigue was defined as a decrease in the ability of the neuromuscular system to produce force during continuous activity. Previous studies have reported that there is a 33–55% decrease in muscle strength in MVIC during fatigue, and a 33% reduction in muscle strength as the fatigue criterion of muscle strength (Thomas et al., 2015). Third, the mean frequency (MNF) and median frequency (MF) of the first and last 30 s of the hip abduction fatigue protocol were calculated to ensure fatigue. Previous authors have reported that the decrease in MNF and MF during fatigue are related to isotonic and dynamic muscle contraction. The MFs of different muscles decreased by 4 and 20% in different fatigue tasks (Gayda et al., 2005; Mcmullen et al., 2011; Zaman et al., 2011). The last 30 s MF of the fatigue scheme reduced the MF by more than 4% compared with the first 30 s as the fatigue criterion in our study.

### Data analysis

Electromyography data during the fatigue protocol were processed using custom MATLABTM programs (The Mathworks Inc, Natick, MA, USA). We calculated the MF and MNF during the first and last 30 s of the hip-abduction fatigue protocol to examine this isolated fatigue protocol. First, all signals were filtered using a 20 Hz high-pass and 450 Hz low-pass Butterworth filter design. The 30-s filtered EMG data were divided into six 5-s sections. The power spectra of each section were calculated using a fast Fourier transform (Wang et al., 2019), and then the MF and MNF for the spectrum were identified.

All kinematic and kinetic data of the hip and knee joint were processed using Visual3D software (Visual 3D, C-motion Inc., Germantown, MD, USA). Ankle, knee and hip joint centers were calculated using the coordinates of the static neutral standing trial. The midpoints between the medial and lateral joint markers were defined as the ankle and knee joint centers. The hip joint center was calculated from the ASIS and PSIS markers, which was defined according to a previous study (Bell et al., 1990). All 3D joint angles were referenced as the distal segment relative to the proximal segment with the Cardan sequence of rotations using an X-Y-Z (Greuel et al., 2017) (x = flexion/extension, y = abduction/adduction, z = internal/external rotation). An inverse dynamics approach was used to calculate the joint kinetic data of the hip and knee joint from the GRF and kinematic data (Kadaba et al., 1990). The joint moments were normalized to body mass. We analyzed joint kinematics and moments for the stance phase and normalized them to 101 data points.

Statistical analyses were performed with SPSS Statistics 22.0 to analyze the intergroup and intragroup differences in gait parameters and surface EMG. Demographic data were collected for descriptive statistics and are described as the mean ± standard deviation (SD). The Shapiro–Wilks test was used to check whether the gait parameters and surface EMG data in the non-fatigue and fatigue states were in accordance with the normal distribution. If the differences were normally distributed, we used a paired t test to determine whether hip abductor fatigue significantly affected knee kinematics and kinetics. The Wilcoxon signed rank test was used for the resulting variables whose differences were not normally distributed. We also used an independent sample t test to compare gender differences in all data before and after fatigue. The Mann–Whitney *U* test was used if the difference values did not conform to homogeneity of variance. The significance level for each test was set *a priori* at 0.05.

## Results

In this study, only 15 people met our standard of hip abductor fatigue (10 females, 5 males; age = 24.80 ± 0.79 years, 25.40 ± 1.67 years; height = 161.70 ± 5.85 cm, 172.20 ± 2.39 cm; body mass = 51.20 ± 4.21 kg, 62.60 ± 3.71 kg; body mass index = 19.57 ± 1.06 kg/m^2^, 21.13 ± 1.43 kg/m^2^), providing data for analysis (Supplementary Table 1). All participants were right leg dominant. At the end of fatigue protocols, all participants reported a score of 19 or higher on the Borg RPE 6–20 scale.

### Hip fatigue

**FIGURE 3:**
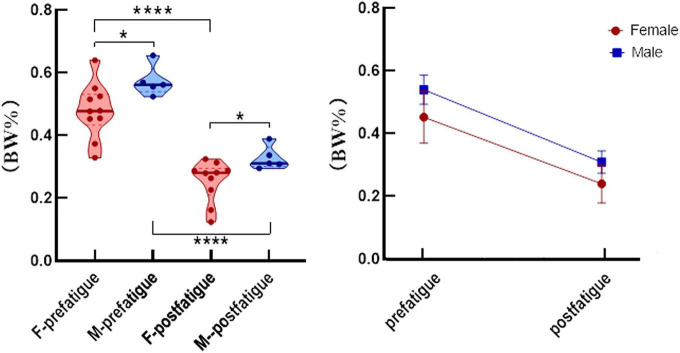
After weight normalization, the average isometric hip strength (mean and standard error) was significantly different after fatigue in men (blue) and women (red). *P < 0.05, ****P < 0.0001.

**FIGURE 4:**
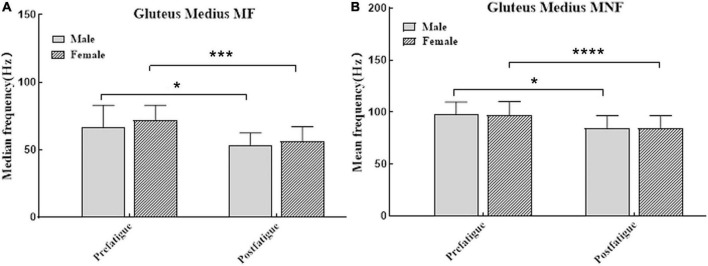
**(A)** The median frequency (mean and standard error) were significantly different after fatigue in men (dark bars) and women (bar with oblique line). **(B)** The mean frequency (mean and standard error) were significantly different after fatigue in men (dark bars) and women (bar with oblique line). *P < 0.05, ***P < 0.001, and ****P < 0.0001.

The average time for participants to fatigue was 684.867 ± 382.422 (s), compared to 689.9 ± 372.453 (s) for men and 684.8 ± 446.922 (s) for women. There was no significant difference between the two groups (*P* > 0.05). Peak isometric hip-abduction strength and median muscle frequency and mean muscle frequency data before and after the strength testing are summarized in Table 1. At the end of the fatigue test, the muscle strength of the participants decreased (95% CI: 10.93–14.57; *P* = 0.000). Peak isometric hip abduction strength decreased by 43% (95% CI: 38.50–47.11; *P* = 0.000) in men and 46% (95% CI: 39.96–54.21; *P* = 0.000) in women. After weight normalization, the hip abductor strength of male participants was significantly higher than that of female participants either before or after fatigue (*P* < 0.05, Figure 3). SEMG data demonstrated that over the course of the fatigue protocol, MF decreased by 21% (95% CI: 9.29–20.20; *P* = 0.000, Figure 4), and MNF (95% CI: 9.67–18.77; *P* = 0.000, Figure 4) decreased by 15%. However, SEMG data of the GM muscle were not different for men or women (*P* > 0.05, Table 1).

**TABLE 1:** Mean (SD) maximum voluntary isometric contractions, median frequency, and mean frequency values before and after fatigue.

Variable	Women (*n* = 10)				Men (*n* = 5)				*P*-value (Group)	
			
	Pre-fatigue	Post-fatigue	*P*-value	Effect size (*d*), 95% confidence interval	Pre-fatigue	Post-fatigue	*P*-value	Effect size (*d*), 95% confidence interval	Pre-fatigue	Post-fatigue
Maximum Voluntary Isometric Contractions (%BW)	0.480 ± 0.088	0.255 ± 0.065	0.000****	2.908 (0.182,0.267)	0.573 ± 0.049	0.328 ± 0.038	0.000****	5.58 (0.182,0.267)	0.047*	0.040*
mean frequency, (Hz)	97.093 ± 13.047	84.750 ± 11.985	0.000****	0.985 (7.619,17.067)	97.656 ± 12.009	84.750 ± 11.985	0.019*	1.076 (4.840,31.090)	0.594	0.502
median frequency, (Hz)	71.880 ± 11.136	56.460 ± 10.834	0.001***	1.404 (7.955,22.885)	67.000 ± 16.045	53.600 ± 8.837	0.035*	1.035 (1.540,25.260)	0.2541	0.619

BW, Body weight. Effect size (d) is based on standardized differences; *P* > 0.05, *P < 0.05, ***P < 0.001, ****P < 0.0001.

### Changes at the hip

**FIGURE 5:**
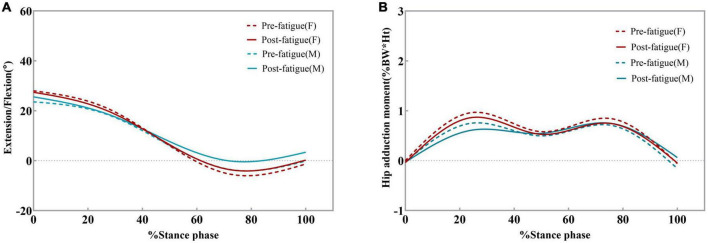
Ensemble average hip kinematics and kinetics for the dominant limb for all male and female participants. Changes in hip flexion angles **(A)** and hip adduction moments **(B)** pre-fatigue and post-fatigue. Moments are given in% bodyweight times height (Ht).

The *post hoc* analysis showed that after fatigue, the hip range of motion (ROM) of participants (*P* < 0.01, Table 2) decreased from flexion to extension (Figure 5). There were significant differences in hip flexion and extension between the sexes before (95% CI: 4.329–11.029; *P* = 0.000, Table 2) and after fatigue (95% CI: 3.965–11.083; *P* = 0.001, Table 2).

**TABLE 2:** Study limb mean (SD) peak kinematic and joint kinetic outcomes from the before and after fatigue gait analyses.

Variable	Women (*n* = 10)				Men (*n* = 5)				*P*-value (Group)	
			
	Pre-fatigue	Post-fatigue	*P*-value	Effect size, 95% confidence interval	Pre-fatigue	Post-fatigue	*P*-value	Effect size, 95% confidence interval	Pre-fatigue	Post-fatigue
The sagittal plane ROM of hip (°)	38.235 ± 2.273	35.418 ± 3.115	0.041**	1.033 (0.151,5.482)	30.556 ± 3.798	27.894 ± 2.751	0.033*	0.803 (0.344,4.980)	0.000***	0.001**
Hip adduction moments (N⋅m⋅kg^–1^)	1.056 ± 0.215	0.977 ± 0.199	0.006**	0.381, (0.029,0.128)	0.815 ± 0.096	0.934 ± 0.238	0.328	–0.656 (–0.416,0.178)	0.035*	0.716

ROM, Range of motion. Effect size (d) is based on standardized differences; *P* > 0.05, *P < 0.05, **P < 0.01, ***P < 0.001.

Figure 5 shows the external hip adduction moments (EHAM) during the stance phase. The EHAM had two peaks at the early and late stance phases. There was no significant difference in the peak value of EHAM before and after fatigue. In female participants, the peak value of EHAM was smaller than that before fatigue (95% CI: 0.029–0.128; *P* = 0.006, Table 2), but no significant difference was found in male participants. This may be due to the small sample size of male participants affecting the test results. There were no significant differences in the peak EHAM between the sexes after fatigue.

### Knee kinematics

**FIGURE 6:**
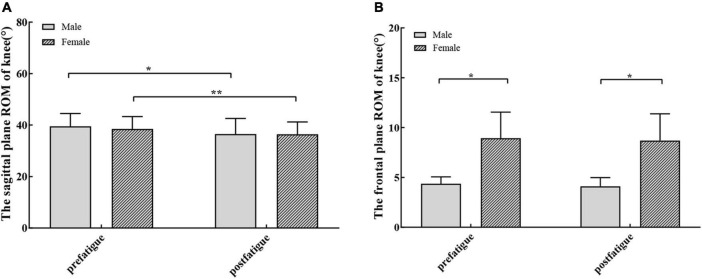
**(A)** The knee sagittal plane range of motion (mean and standard error) were significantly different after fatigue in men (dark bars) and women (bar with oblique line). **(B)** There were no significant differences in frontal plane range of motion (mean and standard error) after fatigue. There were significant differences in the frontal plane range of motion among different sexes before and after fatigue. *P < 0.05, **P < 0.01.

**FIGURE 7:**
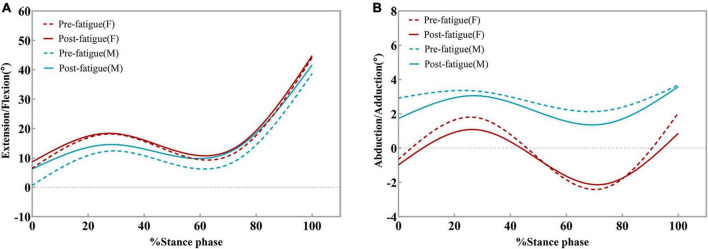
Ensemble average knee kinematics for the dominant limb for all male and female participants. Changes in knee flexion angles **(A)** and knee adduction angles **(B)** pre-fatigue and post-fatigue.

*Post hoc* analysis showed that the range of flexion and extension of the knee joint decreased significantly after fatigue (*P* < 0.0001). The average decrease after fatigue was 2.912° (95% CI: 0.939–4.885; *P* = 0.009, Figure 6) for women and 2.994° (95% CI: 0.305–5.683; *P* = 0.037, Figure 6) for men. The peak knee flexion angles were similar during the pre-fatigue and post-fatigue walking conditions (*P* > 0.05, Table 3 and Figure 7). There were significant differences in the peak knee flexion angle between the sexes before fatigue. The peak flexion angle before fatigue of female participants was significantly greater than that of male participants (95% CI: 1.332–15.530; *P* = 0.044, Table 3).

**TABLE 3:** Study limb mean (SD) knee joint kinematic outcomes from the before and after fatigue gait analyses.

Variable	Women (*n* = 10)				Men (*n* = 5)				*P*-value (Group)	
			
	Pre-fatigue	Post-fatigue	*P*-value	Effect size, 95% confidence interval	Pre-fatigue	Post-fatigue	*P*-value	Effect size, 95% confidence interval	Pre-fatigue	Post-fatigue
Peak knee flexion angle (°)	45.742 ± 5.748	43.944 ± 5.567	0.170	0.318, (–0.930,4.526)	39.412 ± 3.586	42.029 ± 7.305	0.427	0.455, (–10.839,5.605)	0.044*	0.580
Peak knee adduction angle (°)	4.952 ± 3.624	4.410 ± 4.109	0.255	0.140, (–0.467,1.551)	5.223 ± 1.733	4.592 ± 1.177	0.159	0.426, (–0.382,1.644)	0.878	0.925
The sagittal plane ROM of knee (°)	38.963 ± 4.886	36.051 ± 4.530	0.009**	0.618, (0.939,4.885)	39.516 ± 5.009	36.522 ± 6.060	0.037*	0.539, (0.305,5.683)	0.841	0.868
The frontal plane ROM of knee (°)	8.935 ± 2.617	8.696 ± 2.688	0.959	0.090, (Wilcoxon signed rank test)	4.365 ± 0.690	4.103 ± 0.875	0.121	0.333, (–0.109,0.634)	0.002**	0.003**

ROM, Range of motion. Effect size (d) is based on standardized differences; *P* > 0.05, *P < 0.05, **P < 0.01.

After the hip abductor fatigue protocol, no significant difference was found in the frontal plane knee ROM (*P* > 0.05, Table 3 and Figure 7). There were significant differences in the frontal plane ROM between the sexes before fatigue (95% CI: 1.954–7.187; *P* = 0.002, Table 3) and after fatigue (95% CI: 1.884–7.302; *P* = 0.003, Table 3). Both before and after fatigue, the frontal plane ROM of female participants was significantly greater than that of male participants. Participants displayed no differences in the peak knee adduction angle before and after fatigue, and there was no difference between the sexes (*P* > 0.05, Table 3).

### Knee kinetics

**FIGURE 8:**
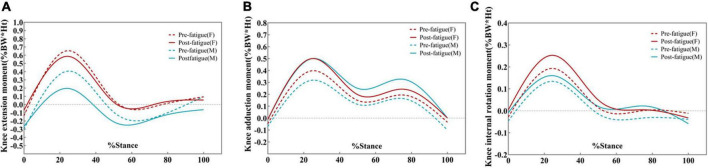
Ensemble average knee kinetics for the dominant limb for all male and female participants. Changes in knee extension moments **(A)**, knee adduction moments **(B)**, and knee internal rotation moments **(C)** during pre-fatigue and post-fatigue. Moments are given in % bodyweight times height (Ht).

**FIGURE 9:**
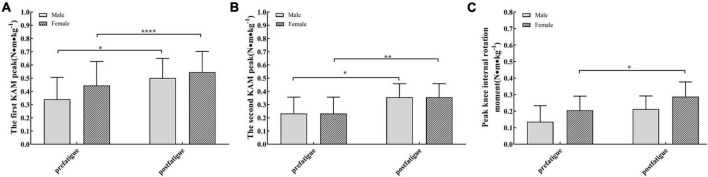
**(A)** The first peak of external knee adduction moment in female (bar with oblique line) and male participants (dark bars) was significantly different after fatigue. **(B)** The second peak of external knee adduction moment was significantly different after fatigue in men (dark bars) and women (bar with oblique line). **(C)** The peak knee internal rotation moments (mean and standard error) were significantly different after fatigue in men (dark bars). *P < 0.05, **P < 0.01, ****P < 0.0001.

Both male and female participants displayed no differences in the peak external knee extension moment after fatigue. However, there was a significant difference between the sexes before (*P* = 0.043, Table 3) and after fatigue (*P* = 0.037, Table 3). Regardless of before or after fatigue, the peak knee extension moment of female participants was higher than that of male participants. Figure 8 shows the external knee adduction and extension moments during the stance phase. The EKAM had two peaks at the early and late stance phases, and the maximum EKAM was observed at the early stance phase. On the coronal plane, the peak values of the first (95% CI: –0.165, –0.077; *P* = 0.000) and second (95% CI: –0.114, –0.043; *P* = 0.000) adduction moments of the knee were significantly increased after fatigue. Female participants demonstrated 22.7% (0.101 N⋅m/kg) greater first knee-adduction moments peak (95% CI: –0.134, –0.067; *P* = 0.000, Table 4 and Figure 9) and 21.1% (0.056 N⋅m/kg) greater second knee-adduction moments peak (95% CI: –0.086, –0.028; *P* = 0.002, Table 4 and Figure 9) in the post-fatigue condition. Male participants demonstrated 47.4% (0.161 N⋅m/kg) greater first knee-adduction moments peak (95% CI: –0.308, –0.014; *P* = 0.039, Table 4 and Figure 9) and 53.0% (0.123 N⋅m/kg) greater second knee-adduction moments peak (95% CI: –0.226, –0.019; *P* = 0.030, Table 4 and Figure 9) in the post-fatigue condition.

**TABLE 4:** Study limb mean (SD) knee joint kinetics outcomes from the before and after fatigue gait analyses.

Variable	Women (*n* = 10)				Men (*n* = 5)				*P*-value (Group)	
			
	Pre-fatigue	Post-fatigue	*P*-value	Effect size, 95% confidence interval	Pre-fatigue	Post-fatigue	*P*-value	Effect size, 95% confidence interval	Pre-fatigue	Post-fatigue
The first KAM peak (N⋅m⋅kg^–1^)	0.444 ± 0.182	0.545 ± 0.157	0.000****	–0.594, (–0.134, –0.067)	0.340 ± 0.166	0.501 ± 0.148	0.039*	–1.024, (–0.308, –0.014)	0.302	0.612
The second KAM peak (N⋅m⋅kg^–1^)	0.265 ± 0.113	0.321 ± 0.106	0.002**	–0.511, (–0.086, –0.028)	0.232 ± 0.124	0.355 ± 0.103	0.030*	–1.079, (–0.226, –0.019)	0.620	0.567
Peak external knee extension moment (N⋅m⋅kg^–1^)	0.779 ± 0.221	0.800 ± 0.232	0.733	–0.092, (–0.160,0.117)	0.519 ± 0.190	0.542 ± 0.115	0.838	–0.146, (–0.317,0.271)	0.043*	0.037*
Peak external knee abduction moment (N⋅m⋅kg^–1^)	–0.133 ± 0.106	–0.098 ± 0.062	0.017*	–0.403, (Wilcoxon signed rank test)	–0.203 ± 0.145	–0.130 ± 0.133	0.043*	–0.525, (Wilcoxon signed rank test)	0.221	0.528
Peak external knee internal rotation moment (N⋅m⋅kg^–1^)	0.205 ± 0.086	0.288 ± 0.089	0.010*	–0.948, (–0.140,–0.026)	0.136 ± 0.097	0.213 ± 0.079	0.288	–0.870, (–0.252,0.098)	0.178	0.136

ROM, range of motion. Effect size (d) is based on standardized differences; *P* > 0.05, *P < 0.05, **P < 0.01, ****P < 0.0001.

The peak value of the knee abduction moment after fatigue was also higher than that before fatigue (*P* < 0.05). Peak external knee abduction moments were 26.3% (0.035 N⋅m/kg) greater in male participants after the fatigue protocol (*P* = 0.017, Table 4 and Figure 9). Female participants demonstrated 36.0% (0.073 N⋅m/kg) greater peak knee-abduction moments in the post-fatigue condition (*P* = 0.043, Table 4 and Figure 9). On the horizontal plane, the peak value of the internal rotation moment is also larger while walking after fatigue (*P* < 0.001, Figure 9).

## Discussion

Hip-muscle weakness has been implicated as a risk factor for ACL injuries, PFPS, and KOA (Hewett et al., 2005; Baldon et al., 2009). There is a growing amount of evidence supporting the influence of hip muscle weakness, as well as changes in lower limb mechanics on the knee joint, which may lead to injuries. The aim of our study was to induce hip abductor weakness through prolonged exertion and then to test injury-related changes in knee kinematics and kinetics. After the fatigue protocol, the peak hip abduction strength of healthy male and female participants decreased by 43 and 46%, respectively. The effect size for the change in strength after the protocol suggests the validity of the isolated fatigue protocol for this study. We observed that after the fatigue protocol of the hip abductor, the decreased hip abductor strength led to a decrease in the sagittal plane ROM of the knee and hip joint and an increase in the external adduction moment at the knee.

### Hip abductor fatigue

The simplest way to determine muscle fatigue is to measure individual load changes during the completion of an exercise task, such as the maximum level of voluntary isometric contraction. This is a visual manifestation of muscle fatigue, the “failure to maintain required or expected force” (Edwards, 1981). Previous studies have reported a 46% (Pohl et al., 2015) and 43% (Patrek et al., 2011) decrease in hip abductor strength, respectively, after the hip abductor fatigue protocol. We observed that the peak hip abduction strength of participants decreased by 44%. After body weight standardization, the hip abductor strength of male participants was significantly higher than that of female participants before and after fatigue. The risk of KOA, PFPS, and ACL injuries in women is significantly higher than that in men (Boling et al., 2010; Frank et al., 2017). Previous studies have shown that the weaker abductor muscles in females may be related to the increase in knee coronal movement and the risk of knee joint injury (Ro et al., 2017). Our research may prove this point to some extent.

Gluteal medius EMG data were recorded during the first 30 and 30 s after the fatigue protocol, providing further objective evidence for hip abductor fatigue caused by the protocol. Most of the literature on the objective performance of muscle fatigue has reported that the variation in muscle fiber propagation velocity will affect the power spectrum of EMG signals (Molinari et al., 2006). During fatiguing isometric contractions, MF mainly reflects the change in conduction velocity (CV) of the active motor units (Mus). From the beginning to the end of muscle fatigue, the high-frequency components of CV are reduced due to the tissue low-pass filtering effect causing the sEMG spectrum to shift to lower frequencies (Zhang et al., 2018). Decreases in MNF and MF are reliable electromyographic indications of local muscle fatigue (Molinari et al., 2006; Zhang et al., 2018). In previous studies, the same hip abduction fatigue protocol recorded 8.6% (Patrek et al., 2011) and 10% (Kogi and Hakamada, 1962) decreases in MF. In our study, we recorded a decrease of 13.4% (female) and 18% (male) in MNF and 21% (female) and 20% (male) decreases in MF. We believe that these EMG changes, coupled with a 43% reduction in peak hip abductor strength and a Borg rating of perceived exertion values of 19/20 for the last 30 s of the fatigue protocol, provide sufficient evidence of hip abductor fatigue.

### Kinematic and kinetic data

Studies have shown that hip abductor weakness is associated with increased hip adduction during dynamic weight-bearing activities in female athletes (Dierks et al., 2008; Willson and Davis, 2008). However, we did not find a difference between the peak hip abduction angle and coronal plane hip ROM. This may be because a decrease in hip abductor strength appears to have little effect on lower limb kinematics: GM has only 20–70% activation during the stand phase of normal walking (Zacharias et al., 2019). Therefore, the requirements of this task may not be sufficient to cause changes in the kinematics of the coronal plane. Tasks that place greater demands on hip abductor may yield greater GM activation and larger kinematic effects than those shown in this investigation. Henriksen et al. (2009) reported that reduced function of the hip abductor resulted in a decrease in peak hip and knee extension angles during gait. In our study, there was no significant difference in the peak flexion angle of the hip and knee joint during walking after fatigue. We observed that the sagittal plane ROM of the hip and knee joint was smaller than that before fatigue.

In our study, there were no associated changes in EHAM after experimentally reducing hip abductor strength. This result is consistent with the results of regression analyses by Rutherford and Hubley-Kozey (2009), who found that posterior abductor strength did not explain variability in EHAM. In contrast to our results, Henriksen (Gayda et al., 2005) observed that after intramuscular injection of hypertonic saline, reduced hip-abductor function was accompanied by a decrease in EHAM during walking. The difference in results may be attributed to the type of intervention. The biomechanical changes they observed after injection could stem from analgesic gait adaptation. Chang et al.’s (2005) study found that a greater baseline hip adduction moment protected against ipsilateral medial OA progression from baseline to 18 months. Chang et al. (2005) postulated that the lower EHAM was due to the decrease in hip abductor strength, resulting in greater contralateral pelvic drop and EKAM. In our study, there was no significant difference in EHAM after fatigue. However, the reduction in hip-abductor strength results in greater EKAM.

One previous study adopted a probabilistic modeling approach to explore the effect of hip abductor weakness on normal gait (Valente et al., 2013). This model demonstrated that weakness of the hip abductor muscles mainly affected hip and knee contact forces during normal walking. There were greater increases in the peak knee joint loads than in loads at the hip. One possible explanation for the lack of changes in hip kinematics is that the magnitude of the induced hip-abductor weakness in our study was insufficient to evoke changes. According to the authors, the greater increase in peak knee load is due to compensation for the hip abductor by the muscular system. Unfortunately, we did not measure the EMG activity in the knee-spanning muscle (rectus femoris and biceps femoris) to support or oppose this. This is a limitation of our study design.

The EKAM based on inverse dynamics analysis is the most commonly used dynamic parameter to reflect the medial load of the knee joint. There is evidence that the peak EKAM is positively correlated with disease progression and knee joint pain (Rutherford and Hubley-Kozey, 2009; O’Connell et al., 2016), and a higher peak EKAM is related to radiographic changes in the knee joint structure and cartilage degeneration (Chehab et al., 2014). In asymptomatic people, the presence and severity of medial meniscus tears were also positively correlated with the peak EKAM (Davies-Tuck et al., 2008). The results of our study indicate that hip abductor fatigue leads to an increase in EKAM, which is consistent with the observations of Geiser et al. (2010). However, some studies have come to different conclusions. Pohl et al. (2015) showed that a reduction in the force output of the hip abductor muscles by superior gluteal nerve block injection did not result in a subsequent increase in EKAM. They reduced hip-abductor function *via* superior gluteal nerve block injection. Nevertheless, the proximity of the injection to the superior gluteal nerve and the duration of the drug effect are difficult to control. This may have contributed to the difference in results. Henriksen et al. (2009) observed a decrease in the EKAM peak after a pain-induced decrease in hip abductor function. It is important that Henriksen’s study reduced hip abductor function by inducing a pain response. It is unclear whether the gait adaptation they observed after injection stems from the analgesic gait pattern.

Inevitably, our study has several limitations. First, these results should be viewed with respect to the characteristics of the participants. As this study was conducted on healthy participants, the current results cannot be directly translated to joint dynamics in patients with knee joint diseases. Therefore, we will further expand the sample size and enrich the sample composition in future studies. Second, the reduction in strength associated with fatigue in this study was used to represent hip abductor muscle weakness. It is important that the kinematic and kinetic changes caused by muscle fatigue may be different from those caused by muscle weakness. Muscle weakness occurs over a long period of time, and compensation patterns can be developed to address this weakness. This adaptive gait change cannot be detected in our study design. It is necessary to further study the effect of hip abductor weakness on knee joint mechanics. Third, we should record EMG activity in more knee and hip muscles to confirm the fatigue compensation strategy.

## Conclusion

We examined knee kinematics and kinetics and hip abductor EMG response to a hip-abduction fatigue protocol in healthy people. Our results revealed that after the fatigue protocol, the sagittal plane knee ROM decreased, and the EKAM increased. From a clinical perspective, significant weakness of the hip abductor muscles exists in people with KOA, PFPS, and ACL injury. The hip abductor muscle plays an important role in gait control and step-to-step symmetry in normal people. Therefore, evaluating hip abductor strength and providing intensive training for patients with muscle weakness is an important part of preventing knee-related injuries.

## Data availability statement

The original contributions presented in the study are included in the article/Supplementary material, further inquiries can be directed to the corresponding authors.

## Ethics statement

The study was reviewed and approved by the Ethics Committee of Huadong Hospital (No. 2020K079). The patients/participants provided their written informed consent to participate in this study.

## Author contributions

JZ and BA conceived and designed the experiments. YT and XZ acquired and analyzed the data. YT wrote this manuscript. YL and MY checked the manuscript. All authors read and approved the final manuscript.

## Abbreviations

ACL Injuries: anterior cruciate ligament injuries
CV: conduction velocity
EHAM: external Hip adduction moment
EKAM: external Knee adduction moment
GM: gluteus medius
GRF: ground reaction force
KOA: knee osteoarthritis
MC-KOA: medial compartment knee osteoarthritis
MF: median frequency
MNF: mean frequency
MVIC: maximum voluntary isometric contraction
PFPS: patellofemoral pain syndrome
ROM: range of motion
sEMG: surface electromyography
WA: weight acceptance.
