# Supplementary material for: The effect of hip abductor fatigue on knee kinematics and kinetics during normal gait
Source: Front Neurosci. 2022 Oct 4;16:1003023. doi: 10.3389/fnins.2022.1003023 (PMC9577318; doi:10.3389/fnins.2022.1003023)
Supplement: Supplementary file 1 [file Table_1.DOCX]

**Table S1.Demographic data of participants**

| [Gender](javascript:;) | Number | Age(y) | Height(cm) | Weight(kg) | Body mass(kg/m^2^) |
| --- | --- | --- | --- | --- | --- |
| Male | 5 | 25.40 ± 1.67 | 172.20 ± 2.39 | 62.60 ± 3.71 | 21.13 ± 1.43 |
| Female | 10 | 24.80 ± 0.79 | 161.70 ± 5.85 | 51.20 ± 4.21 | 19.57 ± `1.06 |
